# A qualitative study of speaking out about patient safety concerns in intensive care units

**DOI:** 10.1016/j.socscimed.2017.09.036

**Published:** 2017-11

**Authors:** Carolyn Tarrant, Myles Leslie, Julian Bion, Mary Dixon-Woods

**Affiliations:** a Department of Health Sciences, University of Leicester, Leicester, UK; b Cumming School of Medicine, University of Calgary, Calgary, Canada; c Institute of Clinical Sciences, University of Birmingham, Birmingham, UK; d Cambridge Centre for Health Services Research, University of Cambridge, Cambridge, UK

**Keywords:** United Kingdom, Patient safety, Speaking up, Intensive care units, Qualitative, Healthcare professions

## Abstract

Much policy focus has been afforded to the role of “whistleblowers” in raising concerns about quality and safety of patient care in healthcare settings. However, most opportunities for personnel to identify and act on these concerns are likely to occur much further upstream, in the day-to-day mundane interactions of everyday work. Using qualitative data from over 900 h of ethnographic observation and 98 interviews across 19 English intensive care units (ICUs), we studied how personnel gave voice to concerns about patient safety or poor practice. We observed much low-level social control occurring as part of day-to-day functioning on the wards, with challenges and sanctions routinely used in an effort to prevent or address mistakes and norm violations. Pre-emptions were used to intervene when patients were at immediate risk, and included strategies such as gentle reminders, use of humour, and sharp words. Corrective interventions included education and evidence-based arguments, while sanctions that were applied when it appeared that a breach of safety had occurred included “quiet words”, bantering, public exposure or humiliation, scoldings and brutal reprimands. These forms of social control generally functioned effectively to maintain safe practice. But they were not consistently effective, and sometimes risked reinforcing norms and idiosyncratic behaviours that were not necessarily aligned with goals of patient safety and high-quality healthcare. Further, making challenges across professional boundaries or hierarchies was sometimes problematic. Our findings suggest that an emphasis on formal reporting or communication training as the solution to giving voice to safety concerns is simplistic; a more sophisticated understanding of social control is needed.

## Highlights

• Most opportunities to raise safety concerns may arise in routine clinical work.
• Informal strategies for raising concerns are multiple and often effective.
• Use of strategies varies within and between professional groups and hierarchies.
• Increased focus on effective use of informal strategies of social control is needed.

## 1 Introduction

Much policy focus has been afforded to the role of “whistleblowers” in raising concerns about quality and safety of patient care in healthcare settings (Francis, 2015, NHS Improvement England., 2016). Whistleblowing is, however, only one type of behaviour for raising concerns. It is likely to be deployed reactively, after incidents have taken place or weaknesses have been detected, and only when other efforts to be heard or to take action have been frustrated. Many more opportunities for personnel to identify and intervene in concerns about the quality or safety of patient care are likely to occur much further upstream in the routine interactions of everyday work, though the exercise of “voice” (Morrison, 2011).

Defined as ‘non-required behaviour that emphasises expression of constructive challenge with an intent to improve rather than merely criticise’ (Dyne et al., 2003 p. 109), voice is directed towards others within the workplace, either to peers (‘speaking out’) or supervisors/managers (‘speaking up’) (Liu et al., 2010). Voice behaviour is a form of prosocial and constructive activity (Dyne et al., 2003), motivated by a desire to optimise performance and avoid error or harm. The ability of teams and individuals to speak out when they have concerns, and to accept challenges and input from others, is often seen as critical for promoting safety in high-risk settings (Lyndon, 2006, Orasanu and Fischer, 2008). Yet use of voice may be potentially risky: among other things, it involves challenging others or disrupting the status quo (Liu et al., 2010). People's willingness to speak out is thus highly dependent on their beliefs about perceived efficacy, and whether they think it will have any negative outcomes (Lyndon, 2008, Morrison, 2011). Attempts to address reluctance to use voice have tended to focus on training in more effective communication strategies (such as avoiding mitigated language and using graded assertiveness) (Okuyama et al., 2014), especially in communicating across hierarchies and boundaries (e.g. Brindley and Reynolds, 2011). However, interpreting challenges in exercise of voice as simply problems of communication is insufficient. In this paper, we propose that understanding how to support those who seek to intervene in potentially inappropriate or unsafe behaviour in healthcare requires an understanding of social control.

### 1.1 Social control in a ‘company of equals’

Processes of social control – including the establishment and enforcement of social norms and informal conflict resolution mechanisms – play an important role in regulating behaviour in interdependent groups (Lazega, 2000). Social norms are ‘standards of behaviour […] based on widely-shared beliefs about how individual group members ought to behave in a given situation’ (Fehr and Fischbacher, 2004 p. 185). Group members are, in principle, incentivised to monitor and use informal social sanctions (such as exclusion) against individuals who violate these norms (Sripada, 2005). Sanctions function to re-enforce social norms and deter future violations; they tend to be graduated dependent on the severity and frequency of the violation (Ostrom, 1990). Social control, exercised through the “informal” use of voice and sanctions in response to norm violations, is thus a potentially effective means of identifying and resolving safety concerns without recourse to external parties and formal corrective systems, including whistleblowing.

In a healthcare context, early evidence of the importance of social control emerged from Freidson and Rhea's work, which examined a group of qualified physicians working together in a large US clinic (Freidson and Rhea, 1963). Describing how the physicians informally monitored each other's conformity to professional and social rules and norms, this work identified that “the elements involved in the process by which control may be exercised in this company of equals are fairly unbureaucratic in character”, and included physicians' use of what Freidson and Rhea describe as “punishments”, including the so-called “talking to”. Similarly described in Rosenthal's later work on incompetent doctors in the UK (Rosenthal, 1995), sanctions such as the “terribly quiet chat” were often (though not always) effective.

This work was important in showing that social control may facilitate ongoing monitoring of colleagues’ behaviours and practices and provide the ability to intervene in ways that may minimise burden and conflict. But it also showed that social control has its limitations. Professional and social norms may not always be aligned with goals of quality and safety; respect for professional autonomy may preclude clinicians from challenging others; and the system is reliant on individuals internalising professional and social norms and being responsive to social sanctions (Dixon-Woods et al., 2011).

This literature offers an intriguing hint that by the time healthcare personnel resort to whistleblowing, a failure of informal social control has already occurred. There is, however, little empirical evidence of what features of social control are used, and to what effect, in the context of the modern healthcare environment; Freidson and Rhea's work predates the era of multidisciplinary teams, and Rosenthal's work predates much of the patient safety and quality movement and major policy shifts.

In this paper, we focus on processes of social control in everyday clinical practice within multidisciplinary healthcare environments, outside the formal reporting processes for dealing with incidents and poor practice. We examine the types of challenges and sanctions used in these environments to prevent or correct behaviours or actions that may pose risks to patient safety, and we reflect on the role and limits of social control in promoting safe and high quality care.

## 2 Methods

This paper reports data from an ethnographic study of a national programme (Dixon-Woods et al., 2013) to reduce central venous catheter bloodstream infections (CVC-BSIs) in intensive care unit (Bion et al., 2013). The interventions introduced as part of the programme included a checklist designed to promote adherence to good practice and facilitate personnel in challenging poor practice in relation to catheter insertion, as well as a number of non-technical interventions targeting safety culture and multi-disciplinary communication about safety.

Across 19 purposively sampled adult ICUs across nine different hospital trusts, authors CT and ML conducted ∼910 h of ethnographic fieldwork. We observed day-to-day interactions around catheter maintenance and care, including how personnel responded when they had concerns about others' practices or aspects of patient safety. We recorded instances of individuals challenging or sanctioning others in response to behaviour or actions that could potentially risk patient safety. Face-to-face interviews were conducted with 98 ‘shop floor’ personnel, including 34 consultants (equivalent to attendings in the US); 14 doctors in training, usually referred to as “junior doctors”, including registrars (residents in the US) and physicians in their foundation years (similar to interns in the US); 28 senior nurses (e.g. nurse managers); 8 staff nurses (qualified registered nurses); and 14 infection prevention/microbiology personnel (specialised nurses and physicians). We questioned participants about their feelings and experiences of responding to concerns about poor practice, and of challenging and sanctioning others. Written informed consent was obtained prior to interview; for observations, people were informed and verbal permissions obtained where possible. Ethical approval for the study was obtained from Berkshire Research Ethics Committee [Ref: 10/H0505/2].

Interviews and fieldnotes were transcribed and anonymised. Analysis of data was based on the constant comparative approach (Charmaz, 2006). Initially, “open codes” were generated, representing the significance of sections of text. These were then incrementally grouped into organising categories or themes. Categories were modified and checked constantly in order to develop a coding frame, which was programmed into NVIVO software and used to process the dataset systematically.

### 3 Findings

We identified a range of informal processes of social control, including challenges – principally in the form of pre-emptions – and post-hoc informal corrective interventions and sanctions of various kinds that were routinely deployed as part of day-to-day work. Though mostly effective in promoting positive patient safety behaviours, some unwanted consequences of these processes were also evident.

### 3.1 Pre-emptions

The major form of prospective challenge that we identified was that of the *pre-emption*, which we defined through our analysis as challenges or corrective interventions that were used to prevent error or poor or risky practice in real-time. Pre-emptions were used by colleagues to alert others when they were at risk of violating social norms or accepted routines of safe practice, and functioned to sensitise them to the need to alter their behaviour. They were of different types, including suggestions and offers of advice or help, questions, reminders, sharp words, education, and banter.

What we termed “gentle” pre-emptions were often framed in very polite, mitigated language (O'Hare and Roscoe, 1990): for example, they took the form of questions, and used qualifications such as ‘would’ or ‘shall’. These gentle pre-emptions allowed for ‘face-saving’ work (Brown and Levinson, 1987, Goffman, 1955) – in which lapses in practice could be attributed to oversights rather than errors due to lack of care or skill.

*The registrar [resident] was getting ready to put the drug into the patient's central line. She picked up the port [without wiping it with an antiseptic wipe to reduce the risk of infection] and the staff nurse immediately said, “I'll get you a wipe for that”. (Fieldnotes, Unit 15)*

By using pre-emptions, individuals were able to prompt others to correct their behaviour in an unthreatening way that averted risk of escalation, consistent with research showing that professionals may ‘shy away from face-to-face conflicts as well as direct and coercive exercise of power’ (Lazega, 2000).

*One of the operating room staff said to the consultant [attending physician], “Do you want the adrenalin turned on?” the consultant said, “Yes, yes” in a confident and confirmatory way. The [staff member] said, “It's just that it was blocked, turned off”, and the consultant said, in a much quieter tone, almost to himself, “Yes, an oversight”. (Fieldnotes, Unit 8)*

Gentle pre-emptions were used both vertically (from those lower in the hierarchy to those above) and horizontally (peer-to-peer). They also occurred across professional boundaries, for example between nurses and physicians. In this context, hierarchical positions were not straightforwardly a matter of different professions having different status: senior, experienced and long-standing ICU nurses were, in many of the contexts we observed, powerful individuals who had considerable influence in relation to physicians (especially, but not only, those who had not reached consultant status); less experienced or more junior nurses, such as staff nurses were, however, less powerful. Thus, gentle pre-emptions, though often effective, were a relatively weak form of challenge and, when deployed in contexts of unequal power, were often easy to rebuff.

*The registrar was still trying to put a central line in the jugular, and the ungowned consultant moved around to the patient's head to have a look. The staff nurse said to the consultant, “Oh, do you want to put a gown on”, and she got a pack out and had it in her hands. She was offering it towards the consultant. The consultant said, “No no no no”. The nurse said to him, “Well I'll put the gown on the edge of the trolley here, and I'll just leave it here if you decide you need it”. She wasn't successful in getting the consultant to put the gown on. (Fieldnotes, Unit 14)*

‘Bantering’ and the use of humour were alternative approaches to making gentle pre-emptions. These strategies were commonly used between those whose position in the hierarchy was close, e.g. a consultants to experienced registrars, or nurses (of most grades) and junior doctors. Bantering in these contexts was usually effective so long as the challenge was given and taken in good spirit; in these instances, humour softened the challenge, enabling it to be made without being seen as an assertion of power or an affront to disciplinary status or expertise.

*The registrar put the sterile packs on top of the trolley, but without removing the plastic wrapping that keeps the trolley [cart] clean between uses. The [staff] nurse was laughing at him, said, “Oh are you trying to keep the trolley clean”, and the registrar kind of put on a stupid voice, said “Is it not okay?” The nurse said to him, “No it's fine”, sarcastically, and the registrar said back jokingly, “Oh well you know I can always say that <nurse's name> said it was fine so I'll be okay”, and they laughed about it. And actually, before the registrar took the trolley into the patient's bedside, he did take the plastic wrapper off the trolley as he was supposed to, and put the pack straight onto the top of the trolley. (Fieldnotes, Unit 11)*

Not all pre-emptive challenges were gentle nor was humour used: sometimes they took the form of ‘sharp words’ or insistence. These more pointed interventions were typically used when the individual making the challenge perceived an imminent risk of harm to a patient.

*At one point [during a central line insertion by a registrar] the anaesthetist consultant actually quite sharply said, “Stop”. The registrar had in fact pierced the artery. (Fieldnotes, Unit 6)*

Sharp words drew attention to risks and real or anticipated mistakes; they were aimed at diverting immediate harm and getting the job done safely. They functioned as deterrents in part by averting a course of action that might harm or lead to further harm. Although they were individual corrections, they were, by necessity, often made in public – in view of other professionals and patients. This use of voice thus made risks visible to others. Like gentle pre-emptions, though in a somewhat different way, sharp words displayed norms about what was acceptable or the right thing to do, but did so in a way that could be seen as threatening, potentially inducing the sense of discomfort or shame that comes from being reprimanded. Sharp words were thus sometimes accompanied by an apology – possibly to mitigate against being seen as an overt display of power or aggression.

*The junior doctor moved in to touch the patient, without putting gloves on or a gown. The [staff] nurse spotted her doing this and she looked around and said, quite sharply, “Do you want some gloves and an apron?” Then almost immediately said, “Oh, I'm sorry, I didn't mean to bark at you”. (Fieldnotes, Unit 14)*

### 3.2 Corrective interventions and sanctions

Corrective interventions were made ‘after the event’; these were forms of social control that functioned to demonstrate that an error or violation had occurred or that practice had been sub-optimal. The forcefulness of these interventions varied: softer, gentler post-hoc interventions included the use of education and evidence-based arguments to shape behaviour, while mild sanctions involved use of humour. Harder tactics included the ‘quiet word’, public exposure or humiliation, and, at the extreme, ‘telling off’ or ‘bollocking’ (severe or brutal reprimand).

We observed many examples of education to correct behaviour; most involved senior or more experience personnel educating those more junior, and they were low-level and unthreatening – they were based on the assumption that mistakes and violations are an inevitable part of learning.

*The patient had been started on Total Parenteral Nutrition and the consultant looked at the dose and said [to the registrar], “Oh, we really, we don't start them on such a high dose, […] do you know the different types of bags that we use?”, and the registrar said, “No”, and the consultant said, […] “You don't just write TPN here, you have to prescribe the right bags, I'll show it to you later”. (Fieldnotes, Unit 14)*

*[Staff nurse] came in with continence pads […] and said to the senior nurse rather apologetically, “I know we're not supposed to use these for an arterial line insertion, but is it ok, I couldn't find [the right pads].” […] The senior nurse immediately responded by saying, […] “No you are not to use those, the pads contain woodpulp and they have found that we really shouldn't use those when we are putting lines in, because there is a risk of really bad infections because of the woodpulp”. […] Staff nurse said, “Oh you know I didn't know about that” (Observation, Unit 8)*

Referring to or invoking evidence was an approach commonly used by doctors to challenge their peers, or by senior nurses to challenge senior doctors. It was founded in the perception that it difficult for (autonomous) peers to ‘correct’ each other's behaviour unless they had good reason.

*You back up things you do with the evidence, so it comes, not like “Oh, we do it just because I say so”, but “because those two studies have demonstrated benefit, this one shows X, Y, Z, and that's why I think my interpretation of the data is, and that's why I do it the way what I do”. (Interview, consultant, Unit 15,)*

Humour and banter were also used as a post-hoc sanction, just, as reported above, as they were used as a pre-emptive challenge. Use of banter and humour to expose or sanction violations was often very public, and, when used in a context where personnel had collegial working relationships, it allowed for public norm enforcement and deterrence for future violations. One way in which it functioned was as a form of mild shaming and humiliation (a type of “telling off”).

*One of the registrars walked around the corner to where we were doing the ward round, and she had a red gown and some gloves on, which should have been taken off when she left the patient's bed space. […] The consultant turned around and just looked at her in sort of mock horror and said, “Dr [name]!”, but in a kind of jokey way, and she immediately knew what he was referring to. […] She touched her gown and said “oh, don't worry, they're clean, I haven't touched anything, I haven't done anything yet”, and she dashed off (Fieldnotes, Unit 15)*

The use of humour was not always positive, however: it sometimes risked taking on a bullying tone.

*The senior consultant, having moved on from one bed space to the next, turned around to find that there was nobody actually following him. [… Eventually] the junior doctors, which included a female, came over, and the consultant said archly, “It's like going shopping with your wife, you think you're done, she says she's done and you look back after twenty feet of walking and realise she's not done”. The female registrar nodded and pursed her lips into a forced smile. (Field notes, Unit 5)*

We observed three stronger forms of disapproval or reprimand: ‘the quiet word’, humiliation, and ‘telling off’ or ‘bollocking’. These types of interventions were most likely to be used by those higher in professional hierarchies.

The ‘quiet word’ was a private challenge, directed at correcting individual behaviour – usually between peers (senior physicians, senior nurses) and also senior to junior physician. Quiet words tended to come into play when an individual repeatedly failed to adhere to good practice, or was seen to be violating fundamental, ‘taken for granted’ social or cultural norms.

*One of the doctors can be a bit lackadaisical, but if I try to say, “Well, come on, you know, think about what you're doing” and, “Would you do this in theatre?”, and he'll take it on board. (Interview, consultant, Unit 14)*

Exposing and humiliation were stronger public challenges that relied on using social disapproval to bring behaviour in line with good practice or norms. Typically used as an escalative strategy when low-level challenges had failed, they functioned by publicly exposing someone as a norm violator, using shame, embarrassment and humiliation to force compliance with norms and deter others from violation. This tactic was, for example, used by senior nurses to challenge doctors.

*The surgeons […] left the bed space and started to leave the ward, as they left the [senior] nurse in charge shouted across the ward at them, after one of the surgeons, “wash hands, wash hands” and he smiled back at her, smiled slightly wryly and did before he left the ward. (Fieldwork, Unit 11)*

*Certain surgeons coming round the unit I almost had to humiliate them in front of their trainees. And that was very effective because you could have a quiet word [but] they carried on doing it [failing to adhere to hygiene precautions], and then I eventually would just say, “Stop, you're not touching that patient, you haven't washed your hands, you haven't put an apron on, and I don't care what you do on your wards but on here you do as we do”. (Interview, senior nurse, Unit 1)*

A direct and strong sanction was ‘telling off’, where an individual was personally reprimanded (usually by a senior colleague). Though potentially effective, it risked causing distress, inducing feelings of being undervalued, or souring relations between colleagues – particularly when people felt the telling off was unjustified or was based on idiosyncratic norms. “Telling off” occurred mainly within (rather than across) professional hierarchies. We directly observed relatively few challenges between nurses, likely because in the ICUs we observed, nurses largely work on their own with individual patients on a nurse-per-bedspace basis. Nonetheless, some examples of nurse-to-nurse “telling off” were reported to observers.

*“I was told off”, she said “I got a real dressing down. All I was doing was doing what we would do here […] try and take [a patient's level of] sedation down and so on, [I] was doing that over there, and I was pulled up '*cos *I was told that that's not how we do it here, the nurses don't do anything without asking first. […] It was the senior nurse that told me off”. (Fieldnotes, Unit 2)*

For those on the receiving end, this type of sanction suggested that they were careless, poorly motivated, or had deliberately behaved badly. People resented these implications along with the outsider status that went with them, and were keen to protect their identity as ‘good’ and well-motivated professionals.

An extreme form of telling off, ‘bollocking’, was used almost exclusively by senior doctors towards junior doctors. We observed only a few examples of it, but when it did occur it was a display of power that reinforced strong hierarchies and brought behaviour into line through engendering fear and shame. A bollocking left no doubt as to the prevailing norms and expectations, and that failing to adhere to them could have negative consequences.

*[At the end of the handover], before the door closed behind the last doctor leaving the room the senior consultant looked at [registrar], who was still sitting there with the paper in his hand, and said [loudly], “You've been here for six months, the handovers need to get much tighter. You've been doing this for long enough, you need to get with the programme”. (Field notes, Unit 1)*

There was some evidence of graduation of challenges and sanctions if lower-level efforts failed to bring about change. One method for informal escalation involved calling on a more senior colleague to intervene. This was seen as a last resort, but as a useful supplement to more informal mechanisms in the case of individuals who were unresponsive to lower levels of social control. Nurses in particular felt that being able to call on doctors’ own disciplinary hierarchy to impose harder sanctions could be highly effective.

*[Senior nurse] said there was a time when they were having problems with a particular doctor who wasn't hand washing and […] they'd try to remind him and suggest to him but he still wasn't doing it, and so she decided to contact [medical director] and just let him know of the problem. She said the next day when the doctor came in, he virtually stripped off and showered. (Fieldnotes Unit 11)*

### 3.3 Influences on challenging and sanctioning

Those in the study sometimes found it hard to make effective challenges. The quality of local relationships, the nature of professional hierarchies, boundaries, and identities, as well as motivation to avoid conflict and preserve harmony (or the appearance of it), all strongly influenced willingness and ability to use mechanisms of social control. For example, personnel with infection control responsibilities reported that they recognised the tension between challenging others for their hygiene and infection control practices, and the need to maintain good cooperative relationships with their colleagues in order that they would engage with infection control programmes and activities. This tension was felt particularly by infection control staff based externally to ICUs, and sometimes resulted in their choosing not to challenge in order to avoid damaging their relationships with ICU personnel.

*While we are with the second patient [infection control nurse] whispered to me “I can see a couple of the doctors have got their watches on” [against infection control policy]. She said “I won't pull them up for it while I'm here collecting the data, I don't want them to see us as constantly policing them or nit-picking. Sometimes I just have to turn a blind eye. […] It's important we have a good relationship with the staff so they welcome us in and take on board our data and work with us.” (Observation, Unit 1)*

Using forms of social control such as pre-emptions and sanctions was easier, and more likely to be well received, in contexts where hierarchies were relatively flat and individuals had established good working relationships.

*At [this unit] we, as nurses we are very autonomous, […] we challenge decisions, […] and I think as well it's a sort of a mutual relationship, whereby actually consultants now ask the nursing staff is there anything else you would like to discuss. […] We are very friendly, and so people are not afraid to ask questions. […] However inexperienced or experienced you may be, you know, we've got limitations or we've got weak points, so people are not afraid [to speak up] (Interview, senior nurse, Unit 17)*

*Most ICU nurses do their course now, so you feel you've got the knowledge behind you, and the training behind you, and that you know, particularly the junior doctors when they're coming up, never worked in an ICU, you feel that your knowledge is as good as theirs, and you feel quite happy to challenge them, and you've seen bad practice over the years, that you think “not on this unit, not here, not today, not now”(Interview, nurse, Unit 14)*

Though some nurses reported that they were comfortable with challenging physicians, some physicians reported that direct challenge in the other direction was potentially uncomfortable, in part because of a fear that doctor-to-nurse challenge might be interpreted as bullying.

*I think all of us would feel a bit more careful about the way we did it, because I think, you know, you've got to recognise […] doctors are still probably, have a more authoritative role than nurses, that's still, that power differential is still there. […] I think if you're in a more senior position, you know, you'd be more cautious about telling a nurse actually than the other way around these days, that's my feeling. I certainly would find it much easier to challenge one of my medical colleagues than I would a nurse (Interview, registrar, Unit 15)*

More broadly, in the absence of cultural support, for example when relationships were not collegial in nature or when previous attempts to challenge had been rebuffed or poorly received, use of many forms of social control was more difficult. In some hospitals in our study, for example, the idea of nurses challenging doctors was unthinkable.

*The senior nurse said how difficult it is for the nurses over at [hospital], how the consultants […] don't listen to the nurses and they shout at the nurses. It's going to be really hard for nurses to challenge them. (Field notes, Unit 2)*

*It does take quite a level of assertiveness on the part of the nurse to be able to openly challenge a doctor, it does depend on the doctor, and it depends on the nurse and it depends on the rank and the age and experience of the nurse and the doctor. (Interview, nurse, Unit 1)*

Accordingly, nurses were sometimes anxious about intervening in a doctor's actions and about the consequences of their challenge, and were not always successful in these interventions.

*The nurse had said to [the registrar, before the line insertion had begun], “Are we putting this central line in for the patient's dopamine?” The registrar said “Yes, yes we are”, and the nurse said, “Well he's only on one millilitre [of the drug] […] I'm not sure the patient actually needs a line” [the registrar ignored this statement]. Following a difficult failed central line insertion, […] the nurse said to the registrar, “Well I did say [he didn't need a line] at the start.” (Field notes, Unit 19)*

### 3.4 Problems with mechanisms of social control: reinforcing idiosyncratic and dysfunctional norms

Processes of social control in the form of challenges and sanctions were very often, but not always, positive actions. On occasion, they were oriented towards reinforcing idiosyncratic norms, and, in some settings, the norms of acceptable practice that had been established and reinforced were dysfunctional. In a few settings, some senior individuals – consultants in particular – explicitly enforced their own idiosyncratic norms and expectations with more junior physicians. They sanctioned individuals for failures of adherence – even when these norms conflicted with those of other consultants or were not aligned with evidence-based good practice. Less hierarchically privileged individuals therefore had to ensure they avoided committing what Bosk terms quasi-normative errors (Bosk, 1979), errors that are “idiosyncratic; behaviour that would be formally correct on one service is not so on another” (p64).

*Working with different consultants is that I would say, probably without exception, as registrars we do what the consultant tells us to do. […] So I've put central lines in with just a pair of gloves with the [consultant] who does it like that, because that's how he does it, he won't let you do any other way, and as registrars you're under pressure to do things as the consultant wants you to do them ….[Otherwise it] makes the rest of your day miserable. (Interview, junior doctor, Unit 5)*

The reliance on some forms of social control, such as bantering, was also problematic when local norms were dysfunctional.

*[Registrar] took abuse, jocular abuse, but abuse, from the two consultants […] when in accordance with best practice he requested a surgical cap as part of barrier precautions before inserting a central venous catheter. The first consultant said to the second consultant, “Ah well, tell him to go get his lucky hat”, and the second consultant just laughed, [as he] didn't believe in the evidence for wearing a hat during insertion. (Field notes, Unit 13)*

When wrongdoing is collective it is more likely to become normalised. Further, when dysfunctional norms have been established, observers are less likely to label the behaviour as a wrongdoing, and are more likely to self-censor even if they have concerns (Ashforth and Anand, 2003). We found some examples of where clinicians were aware of expectations of good practice but did not challenge each other for failures of adherence because deviations had become normalised. This occurred, for example, in relation to infection prevention and control practices.

*On this morning's rounds […] everybody stands either on or just inside the red line [marking the patients' bed space]. They can't be bothered with putting an apron on [which they are required to do as an infection control measure to enter the bed space … So] we all stood and then yelled at the patients from outside the red lines. (Fieldnotes, Unit 3)*

## 4 Discussion

Use of processes of social control are a prominent feature of day-to-day functioning in hospitals, as it is in most social environments. In this ethnographic study across multiple intensive care units, we saw healthcare workers of all professional roles use challenges and sanctions to prevent or address actual or potential errors, risky behaviours and norm violations. These informal techniques alerted people to when they were at risk of breaching norms or routines of safe practice; promped individuals to correct their own behaviour; sanctioned those who had violated norms to bring them in to line; and made visible and reinforced norms. There was some evidence of the potential for graduated escalation where low-level challenges had been unsuccessful, with involvement of third parties or formal routes seen as the ‘last resort’. Methods of social control, when they worked well, promoted safety and helped to maintain cooperative relationship. Our observations thus confirm that exercising ‘voice’ is much more than a problem of communication techniques (Orasanu and Fischer, 2008); instead, it is better understood as one important element of the exercise of social control.

Processes of social control are key to the smooth and safe functioning of interdependent healthcare teams, whose members rely on cooperative action around common goals and adherence to norms. Our study identified a diverse and fine-grained set of strategies for influencing the behaviours of others, with evidence that many of these strategies have endured over time: Freidson and Rhea (1963), for example, include in their list of “punishments”, along with the talking to: “various blends of instruction, friendly persuasion of error, shaming the offender, and threatening him [sic] with retalitation”. Our work adds to this in elaborating and extending the forms of sanction that may be used, but also in emphasising the importance of pre-emptive challenge techniques that may be used. Both pre-emptions and post-hoc challenges shared the characteristic that they often involved the public display of norms and exposure of norm violations, perhaps using humour, which helped to shape the behaviour of multiple individuals.

When social control works well it may be very effective in enabling risks to safety to be reduced. It does, however, have limitations. Strategies that rely on peer disapproval to bring people in line with accepted norms are only effective if colleagues monitor each others' behaviour and intervene when needed, and when those who are the target of interventions acknowledge that what they have been doing is ‘wrong’, and care about the disapproval of their peers. Recalcitrant or poorly motivated individuals, as well as individuals who lack skills or who are struggling for other reasons, may be unwilling or unable to correct their behaviour. The ability to exercise social control is not equally available to all: hierarchies and professional boundaries may present barriers (Morrow et al., 2016). In our study, early-career physicians were often the target of processes of social control, consistent with their status as learners, and in line with the well-documented phenomenon of the hidden curriculum (Hafferty and Franks, 1994). Yet they may themselves struggle to challenge others about their practices (Friedman et al., 2015). The same was true of nurses in contexts that were culturally unsupportive. This lack of voice is troubling, since it may result in poorer control of risks to safety, lost opportunities for reflective learning, and inhibited role development.

Further, for all its positives, informal social control has a dark side: it is norm-based, so it involves efforts to bring others into line with accepted norms. These norms are often, but not always, aligned with goals of patient safety and high-quality healthcare. Sometimes, in our study, processes of control were oriented towards maintaining idiosyncratic or dysfunctional norms. Social control might also be seen as acting as a form of ‘containment’ (Lindsay et al., 2012), that can mean that opportunities for wider organisational learning about preventing harm, and organisational intervention are lost (Sujan et al., 2011).

### 4.1 Strengths and limitations

A key strength of our study is the abundance of data we were able to collect across ICUs with diverse characteristics within diverse hospital environments, and from many personnel in different roles, generating important insights into fundamental mechanisms of social control that operate within this acute care environment. Since our observations and interviews were conducted over a period of a few days in each setting, we do not claim to have gained a deep understanding of the dynamics within each setting. We have instead drawn our interpretations from identifying common patterns of interaction, and systematic differences between the approaches of personnel in different roles and positions, across the diverse range of settings we visited. Our observers were non-clinical, meaning that on the one hand they were perceived as non-threatening and non-judgemental by clinicians in the settings studied, but on the other hand meant that they may have missed recognising some opportunities for intervention to prevent or sanction harm. Longer periods of observation and immersion, and a wider range observers, might have generated insight into the less ‘visible’ forms of social control in operation, as well as a more complete understanding of the impact of local context. It might also have enabled further characteristation of interactions within professional groups (e.g. nurse-nurse, ICU physician-microbiology consultant, and so on), which would be of benefit in deepening insight into challenges and sanctions used in groups and hierarchies.

Our study was conducted in ICUs, which have a number of features that make them unique and distinct from other acute care environments. They usually have a much higher staff-patient ratio than other settings; patients are seriously ill (perhaps sedated and variably conscious); interactions between healthcare professionals in ICUs may be different from those settings where patients are more active witnesses and participants. More generally, the nature and pattern of social control in other hospital settings, such as general wards, may differ significantly to that observed within ICUs. Observations in non-ICU settings might have enabled exploration of the possibility that one explanation for the low rate of observations of nurse-to-nurse processes of social control may lie in the different line management and supervisory structures used by nurses, in addition to (or instead of) the specifics of the ICU setting. Though an interview-based study into views of raising concerns in other settings did identify some findings similar to ours (Jones and Kelly, 2014b), further investigation is needed.

Our work makes three important contributions to previous work on social control. First, in contrast to research that has characterised mechanisms of social control among equals (Freidson and Rhea, 1963), our study explores how healthcare personnel regulate others' behaviour across professional boundaries and hierarchies in a company of *unequals*. In these mixed settings, there is less focus on a collegium (Dixon-Woods et al., 2011) whose reputation depends on the behaviour of its members, or on a self-regulating ‘in-group’. Second, much of the literature on social control focuses on the control of ‘deviants’, or ‘bad apples’ (Kerr et al., 2009), yet we found that most social control was not about correcting and controlling bad people, but rather was directed at acts of individual behaviour, and was predicated on the premise that a lot of ‘wrong-doing’ involves a non-deliberate slip, lapse or mistake, or reflects a lack of awareness of local norms. Finally, the literature on social control focuses predominantly on punishment and sanctions (or rewards) in influencing the behaviour of others (Balliet et al., 2011). In this paper we have described a spectrum of strategies, many of them subtle and nuanced, that are brought to bear in exerting influence on others. Mechanisms of social control involve not just graduated sanctions (Ostrom, 1990), but extensive use of pre-emptions and challenges to alert and encourage others to correct their own behaviour.

### 4.2 What are the implications of this work?

Though widely promoted internationally, whistleblowing is reactive. Our study suggests that whistleblowing may be an approach that personnel are likely to draw on when lower-level formal controls have failed; these informal routes for speaking out remain neglected in policy and guidance (Jones and Kelly, 2014a). Our research shows that low-level social control occurring as part of day-to-day functioning in healthcare has a critical role to play in promoting quality and safety. This renewed understanding how safe practice is socially created and controlled proactively is important. Of course, informal mechanisms alone are not sufficient to manage the problem of poor or unsafe practice (Freidson and Rhea, 1963, Stelling and Bucher, 1972). Effective control depends also on having a supportive infrastructure, including the availability of regulatory and/or legal structures that can take over when informal control mechanisms fail and positive local norms (Dixon-Woods et al., 2011). Features of environments likely to promote such norms include a leadership commitment to supporting challenge (and and learning from challenge), an atmosphere of trust and psychological safety (Edmondson, 1999), and clear and consistent messages about “where the line must be drawn between acceptable and unacceptable behaviour” (Gallagher et al., 2013).

Enabling those in hierarchically disadvantaged positions to challenge poor practice in their day-to-day experience, in the face of expectations about them as learners, and in the context of their own developing knowledge and uncertainties (Beament and Mercer, 2016), is a particular issue of concern. Senior personnel may need to take active steps to create a culture in which challenge is welcome, with a focus on shared learning (Edmondson, 2012). Informal means for raising concerns outside the immediate supervision hierarchy are also likely to be important (Carr et al., 2016). Healthcare need to feel safe to challenge each other without fear of rebuff and without damaging relationships; support in how to manage relationships where giving and responding to challenge is problematic may also be of value.

## 4.3 Conclusions

Our study shows why an emphasis on either communication techniques or formal reporting as the solution to resolving problems of unsafe practice is simplistic. The giving of voice to concerns about patient safety in day-to-day clinical care is likely to be best understood as a one of social control.

## Funding

This study was funded by the Health Foundation, charity number 286967, and by Mary Dixon-Woods' Wellcome Trust Senior Investigator Award (WT097899).
